# New Buildings for the Jefferson Medical College of Philadelphia

**Published:** 1892-04

**Authors:** 


### NEW BUILDINGS FOR THE JEFFERSON MEDICAL COLLEGE OF PHILADELPHIA.

The Board of Trustees and the Faculty of the Jefferson Medical College have just completed the purchase of two large lots on Broad street, upon which they will proceed to erect at once, a handsome hospital, lecture-hall and laboratory building. The estimated cost of the building is $500,000. The hospital will be built not only as a suitable building in which to care for the sick and injured, but also will be provided with a large amphitheatre for clinical lectures. The basement of the hospital will be given over to the various dispensaries, each of which will be provided with large waiting and physicians' rooms as well as rooms for direct teaching of the students. By the erection of three commodious buildings, the laboratory where delicate work with the microscope is carried on, will be separated from the college hall where didactic lectures are given and so will be free from any jarring produced by the movement of large classes. Further than this immediately across the street is the Howard Hospital, and on the adjoining corner the Ridgeway branch of the Philadelphia Free Library, which contains all the scientific works belonging to this wealthy corporation. The new site is even more favorably situated in regard to the centre of the city, than the old one at 10th and Sansom streets.

The buildings will be ready for occupancy in the session of '93-'94.

